# The invasive legume *Lupinus polyphyllus* has minor site‐specific impacts on the composition of soil bacterial communities

**DOI:** 10.1002/ece3.11030

**Published:** 2024-02-13

**Authors:** Seyed Abdollah Mousavi, Satu Ramula

**Affiliations:** ^1^ Department of Biology University of Turku Turku Finland

**Keywords:** 16S rRNA amplicon sequencing, plant invasion, rhizobia, soil bacterial community

## Abstract

Plant invasions can have major impacts on ecosystems, both above‐ and belowground. In particular, invasions by legumes, which often host nitrogen‐fixing symbionts (rhizobia), are known to modify soil bacterial communities. Here, we examined the effect of the invasive herbaceous legume *Lupinus polyphyllus* on the alpha diversity and community composition of soil bacteria. We also explored the relationships between these bacterial communities and vegetation cover, the cover of other (non‐invasive) legumes, or the number of vascular plants present. For this, we sampled rhizosphere soil and surveyed vegetation from ten paired sites (uninvaded versus invaded more than 10 years ago) in southwestern Finland, and identified bacterial DNA using 16S rRNA gene amplicon sequencing. The presence of the plant invader and the three vegetation variables considered had no effect on the alpha diversity of soil bacteria in terms of bacterial richness or Shannon and Inverse Simpson diversity indices. However, the composition of soil bacterial communities differed between invaded and uninvaded soils at four out of the ten sites. Interestingly, the relative abundances of the top bacterial families in invaded and uninvaded soils were inconsistent across sites, including for legume‐associated rhizobia in the family *Bradyrhizobiaceae*. Other factors—such as vegetation cover, legume cover (excluding *L. polyphyllus*), number of plant species—also explained a small proportion of the variation in bacterial community composition. Our findings indicate that *L. polyphyllus* has the potential to modify the composition of local soil bacterial community, at least in sites where it has been present for more than a decade.

## Graphical abstract

In our study, we investigated the impact of the invasive herbaceous legume, *Lupinus polyphyllus*, on soil bacterial communities in southwestern Finland. We used 16S rRNA gene amplicon sequencing to analyse soil samples collected from ten paired sites (invaded and uninvaded locations), and examined the potential influence of three vegetation variables (vegetation cover, legume cover, and number of plant species) on the soil bacterial community. Our findings indicate that *L. polyphyllus* has the potential to modify the composition of local soil bacterial community, at least in sites where it has been present for more than a decade.

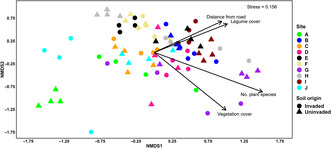

## 1 INTRODUCTION

Plant invasions are recognised as a significant driver of global biodiversity and ecosystem change (Liao et al., 2008; Mack et al., 2000; Vitousek et al., 1997) which can result in the homogenisation of floras and a decline in native plant species richness and overall biodiversity (Daru et al., 2021; Hansen et al., 2021; Hejda et al., 2009; Nowakowski et al., 2018). Invasive plants have been associated with shifts in ecosystem structure both above‐ and belowground (Custer & van Diepen, 2020; Dawson & Schrama, 2016), although the latter has received much less attention (Dawson & Schrama, 2016). Plant invaders have been implicated in alternations of soil microbial alpha diversity and the composition of soil microbial communities (Custer & van Diepen, 2020; Stefanowicz et al., 2016; Torres et al., 2021; Unger et al., 2022), which may have knock‐on effects on plant fitness (Coats & Rumpho, 2014) and the services provided by the ecosystem (Milanović et al., 2020). Nevertheless, the magnitude and direction of soil microbial responses to plant invasions are unclear. A meta‐analysis of the impacts of 23 invasive plant species from different continents on soil bacteria, fungi, and nutrients indicated that plant invasions had highly diverse but limited effects on microbial alpha diversity in soil (Custer & van Diepen, 2020). On the other hand, the results of another meta‐analysis suggested that certain types of invasions‐specifically, of herbaceous plants producing allelopathic substances‐actually increased soil bacterial alpha diversity (Torres et al., 2021). Likewise, studies examining changes in the composition of soil microbial communities in response to plant invasion have also produced mixed results. As an example, a study on three aggressive invasive herbs in a temperate environment revealed species‐specific effects on soil microbial communities (Stefanowicz et al., 2016).

Although many studies have emphasised the essential role of plant species identity in shaping soil microbial communities (Badri & Vivanco, 2009; Costa et al., 2006; Garbeva et al., 2008; Marschner et al., 2004), it is also possible that, on its own, a single (even invasive) plant species has a minimal impact on the soil microbiota. Instead, soil microbial communities might be determined by the local plant community as a whole (Schlatter et al., 2015) or by certain plant functional groups, such as legumes (Dassen et al., 2017). Indeed, several studies have reported a positive relationship between plant species richness and soil microbial richness (e.g. reviewed in Liu et al., 2020). Moreover, soil physical properties (e.g. moisture, compaction), which partially depend on vegetation cover, are also important for the structure of the soil microbiome (Erktan et al., 2020).

Invasions involving leguminous plants serve as interesting model systems for investigating the effects of plant invasion on soil microbial communities. Legumes (Fabaceae) can establish symbiosis with nitrogen‐fixing bacteria (rhizobia), which are the major nitrogen contributors to the terrestrial biosphere (Lindström & Mousavi, 2010; Mousavi, 2016). In this symbiotic relationship, which primarily occurs in root or stem nodules, rhizobia are hosted and fed carbon resources by the legume, and in return rhizobia supply their hosts with nitrogen by reducing atmospheric dinitrogen (N_2_) to biologically available ammonia (NH_4_^+^) (Lindström & Mousavi, 2020; Mousavi, 2016). As a result, when invasive legumes find compatible rhizobia and establish successful symbiosis, they have the ability to dramatically modify soil nutrient cycles (especially nitrogen content) in their new ranges (Keet et al., 2021; Le Roux et al., 2018). Moreover, such alternations in abiotic soil conditions are likely to modify the abundance and diversity of soil microbial communities (Kamutando et al., 2017). For example, the invasion of Australian acacias was found to result in lower alpha diversity and a more homogenous population of soil rhizobia in invaded soils compared to those of uninvaded soils (Le Roux et al., 2018). Changes in soil microbial composition can have major consequences for aboveground communities (Wardle et al., 2004). For plant species associating with mutualistic microbes, the lack of a suitable partner in the soil may limit plant establishment (Simonsen et al., 2017) or considerably reduce growth and resistance to abiotic stress (reviewed in Gopalakrishnan et al., 2015). Moreover, soil bacteria belonging to *Gammaproteobacteria*, *Betaproteobacteria*, and *Firmicutes* promote plant health by suppressing diseases (Berendsen et al., 2012).

*Lupinus polyphyllus* (Lindl.), a legume in the family Fabaceae, is an invasive, short‐lived perennial herb. It is originally from the western USA, but was introduced to many European countries, including Finland, in the 19th century (Eckstein et al., 2023). Like other members of family Fabaceae, *L. polyphyllus* can fix nitrogen through symbiosis with rhizobia. The most common nodule‐forming bacteria found in *L. polyphyllus* belong to the genus *Bradyrhizobium* (e.g. Ramula et al., 2023; Stępkowski et al., 2007, 2018), but members of the genera *Rhizobium* and *Bosea* have also been reported (De Meyer et al., 2011; De Meyer & Willems, 2012). Throughout Europe, invasions of *L. polyphyllus* have been associated with declines in the number of vascular plant species and changes in plant community composition (reviewed in Eckstein et al., 2023). However, to our knowledge, the effect of this widespread invader on soil microbial communities remains to be studied. Here, we investigated the impact of *L. polyphyllus* on soil bacterial communities in southwestern Finland using the amplicon sequencing of the 16S rRNA gene, a commonly used technique for studying the diversity of soil bacterial communities (Mousavi et al., 2022). More specifically, we asked: (i) Does *L. polyphyllus* modify the alpha diversity and composition of soil bacterial communities, and rhizobial abundance in particular (i.e. members of family *Bradyrhizobiaceae*)? (ii) Are the impacts of the invader consistent across sites? (iii) Are soil bacterial alpha diversity and community composition affected by other factors, such as total vegetation cover, the cover of legumes other than *L. polyphyllus*, the number of vascular plant species present? Given the tendency of *L. polyphyllus* to reduce plant species richness locally (Eckstein et al., 2023), we hypothesised that this invader would also be linked to reduced alpha diversity and changes in the community composition of soil bacteria. In particular, we expected to find a higher abundance of nitrogen‐fixing bacteria (family *Bradyrhizobiaceae*) in invaded soils than in uninvaded ones. We also expected that the three vegetation variables would contribute to the composition of soil bacterial communities.

## 2 MATERIALS AND METHODS

### 2.1 Soil and vegetation sampling

From 7 to 10 June 2021, we collected soil samples and surveyed vegetation from ten previously studied pairs of sites, which included ten locations that have been invaded by *L*. *polyphyllus* each matched with a nearby uninvaded location. All sites are close to Turku in southwestern Finland (latitude: 60.357–60.521, longitude: 22.168–22.740). The uninvaded locations were at least 30 m distant from the invaded locations and were often separated from them by a larger road or a forest patch that probably limited further invasion. The distances among the ten sample sites varied from 1.7 to 32.7 km and the sites were located 4–194 m from the closest main road. The sites featured sandy moraine or clay soil and were located in wastelands, road verges, former fields, or forest understoreys, with *L. polyphyllus* being the only member of genus *Lupinus* present. The exact age of the invasions is unknown, but the species has been present in the invaded locations since at least 2010 when we visited the sites for the first time (Ramula, 2014). Currently, populations of *L. polyphyllus* in the invaded locations contain hundreds of individuals (mean cover = 59.7%, range: 2%–96%). The invasion history of the species in southwestern Finland reaches back over a 100 years, with rapid spread occurring in the past few decades. It is likely that annual mowing of road verges and movement of soil masses for infrastructure development have facilitated seed dispersal, as was reported in Sweden (Eckstein et al., 2023) and the same is likely to be true also in Finland. At each site, we took soil samples from the rhizosphere (at a depth of 10–15 cm) of four plants (*L. polyphyllus* in the invaded locations and the dominant species in the uninvaded locations) along a transect, with a distance of six to seven metres between each sample to capture some spatial variation. All sampled plants were either at the vegetative or flowering stage (i.e. seedlings were not sampled). We dug up the sampled plant and used a spade to gently remove soil close to its root system into a plastic ziplock bag. To avoid cross‐contamination, the spade was wiped with ethanol after each sampling event. At all sites, both invaded and uninvaded, grass species (family Poaceae) were among the three most abundant species. While the invaded locations were typically dominated by *L. polyphyllus*, the uninvaded locations were dominated by either grasses (e.g. *Alopecurus pratensis*, *Poa pratensis*; sites A, E, F, G, H and I), *Achillea millefolium* (sites B and D), *Oxalis acetosella* (site C), or *Trifolium repens* (site J). We collected 80 soil samples in total, transported them in a cold box to the laboratory, and preserved them at −20°C for further analyses. In each sampling point, we also recorded total vegetation cover, the cover of *L. polyphyllus*, the cover of other legumes, and the number of vascular plant species in a quadrat of 1 × 1 m.

### 2.2 DNA extraction and PCR amplification

We extracted the genomic DNA of microorganisms from the 80 soil samples with the NucleoSpin Soil kit (Macherey‐Nagel GmbH Co. KG, Duren, Germany) following the steps provided by the manufacturer. The concentration of each extracted DNA sample was measured using the ND‐1000 spectrophotometer (NanoDrop™) and the DNA samples were preserved at −20°C. The methods for the sequencing of the 16S rRNA gene (V4 region) were adapted from those developed for the Earth Microbiome Project (https://earthmicrobiome.org/). To optimise DNA concentration and PCR conditions for our soil samples, we ran trial PCRs and then chose to dilute all DNA samples by a factor of 10. We used the following PCR protocol: 94°C for 4 min, then 30 cycles of 94°C for 20 s, 57.5°C for 15 s, and 68°C for 30 s, using the barcoded V4 primer set 515F/806R. All PCR products were checked on 1.5% agarose gel and the gels were visualised by applying a gel documentation system (BioRad). Then, 5 μL of each PCR product (duplicated) were mixed to create a DNA library. Prepared library samples were sequenced using Illumina Miseq v2 sequencing (2 × 250 bp) at the Finnish Functional Genomic Centre in Turku, Finland.

### 2.3 Bioinformatics and statistical analyses

We processed a total of 11,773,421 raw reads by applying the ASV‐based DADA2 pipeline v.1.22.0 (Callahan et al., 2016) and following the online DADA2 Pipeline Tutorial (1.8) (https://benjjneb.github.io/dada2/tutorial.html) for sequences. After filtering, denoising, merging, and removing chimers, 2,107,724 sequences remained for further analyses (mean = 26,346, range = 8962–39,946). We assigned taxonomic identities to the sequences using the Ribosomal Database Project's (RDP) training set 18 as the 16S rRNA reference dataset (maintained by the DADA2 pipeline). Overall, we identified 11,207 amplicon sequence variants (ASVs) across our soil samples. The ASV and taxonomy tables were imported to RStudio (2022.02.0), which is based on R version 4.1.1. (R Core Team, 2021). We first removed all singletons and non‐bacterial ASVs. To control for differential sequencing depths, we rarefied all samples to the smallest sequencing depth in the data (8956 sequences per sample) for subsequent analyses. We converted absolute abundances into relative abundances and continued our analyses with a total of 9897 ASVs. To describe bacterial alpha diversity, we calculated bacterial richness (i.e. the number of bacterial ASVs per sample); Shannon index, which evaluates the relative abundances of different ASVs with an emphasis on rare species; and the Inverse Simpson index, which weights abundant ASVs more than rare ones. These calculations were carried out using the R packages ‘phyloseq’ (McMurdie & Holmes, 2013) and ‘microeco’ (Liu et al., 2021).

We constructed a linear mixed model (LMM, lmer4::lmer) for the three measures of alpha diversity (bacterial richness, Shannon index, Inverse Simpson index) that used soil origin (invaded, uninvaded), vegetation cover, the cover of other legumes, and distance from the closest main road as fixed explanatory variables, and included site as a random factor. We did not consider the number of vascular species in the same analysis because it was confounded with soil origin (i.e. invaded sites contained fewer plant species than uninvaded ones). We therefore conducted separate LMMs to investigate the relationship between soil bacterial alpha diversity and plant species richness. In these models, we used each of the three diversity measures as a response variable, the number of vascular plant species as a continuous explanatory variable, and site as a random factor. Model assumptions were verified visually from residual plots. To test whether soil origin affected bacterial alpha diversity differentially across sites, we fit different slopes by sites and compared the model AIC to that of the model with a constant slope. Different slopes were not supported in any of the cases (AIC was lower for the models with a constant slope). We evaluated the significance of the fixed variables with an *F* test based on the Kenward‐Roger method (lmerTest::anova; Kuznetsova et al., 2017), and quantified the variance explained by the random variable (site) based on Nakagawa's *R* ^2^, which is suited for mixed models (performance::r2_nakagawa; Lüdecke et al., 2021). The variance contribution was calculated as the difference between the marginal *R* ^2^ (containing fixed effects) and conditional *R* ^2^ (containing both fixed and random effects).

We investigated differences in soil bacterial community composition, including members of *Bradyrhizobiaceae*, with a permutational multivariate analysis of variance (PERMANOVA, vegan::adonis2; Oksanen et al., 2022) based on Bray‐Curtis dissimilarities calculated from the relative abundances of bacterial ASVs. As this analysis does not allow random factors to be included in the model, we used soil origin (invaded, uninvaded), vegetation cover, legume cover (excluding the study species), distance from the road, site, and the interaction between soil origin and site as fixed explanatory variables. The significance of each factor was tested with 999 permutations. We also explored the homogeneity of variances for the bacterial communities between invaded and uninvaded locations to determine whether the plant invader homogenises soil bacterial communities (vegan::betadisper). Finally, we conducted a PERMANOVA to test the association between the relative abundance of bacterial ASVs and the number of vascular plant species (i.e. a separate analysis was again used because plant species number was confounded with soil origin). For the sites where the bacterial communities differed between soil origins (based on visual assessment), we conducted an indicator ASV analysis (indicspecies::multipatt with func = IndVal.g; De Cáceres & Legendre, 2009) to identify taxa that were associated with invaded or uninvaded soils. To assess the effect of geographical distance in structuring soil bacterial communities, we performed a Mantel test that compared the Bray–Curtis dissimilarity matrix, calculated from the relative abundances of bacterial ASVs and the geographical distance matrix, which contained Euclidean distances based on the coordinates of the study locations (vegan::mantel, method = spearman with 999 permutations). We used a non‐metric multidimensional scaling (NMDS) based on Bray‐Curtis dissimilarities (vegan::metaMDS) to visualise bacterial communities in the invaded and uninvaded locations across the ten study sites, and illustrated the effects on bacterial communities of total vegetation cover, legume cover, the number of vascular plant species, and distance from the road by plotting them in the ordination (vegan::envfit). We also visualised bacterial communities in the invaded and uninvaded soils across sites with a hierarchical cluster analysis (unweighted pair group method with arithmetic mean, UPGMA) based on Bray‐Curtis dissimilarities calculated from the mean relative abundances of bacterial ASVs for each location per site (vegan::hclust). All visualisations were created using the R packages ‘ggplot2’ (Wickham, 2016) and ‘ggpubr’ (Kassambara, 2022).

## 3 RESULTS

### 3.1 Soil bacterial alpha diversity

**FIGURE 1:**
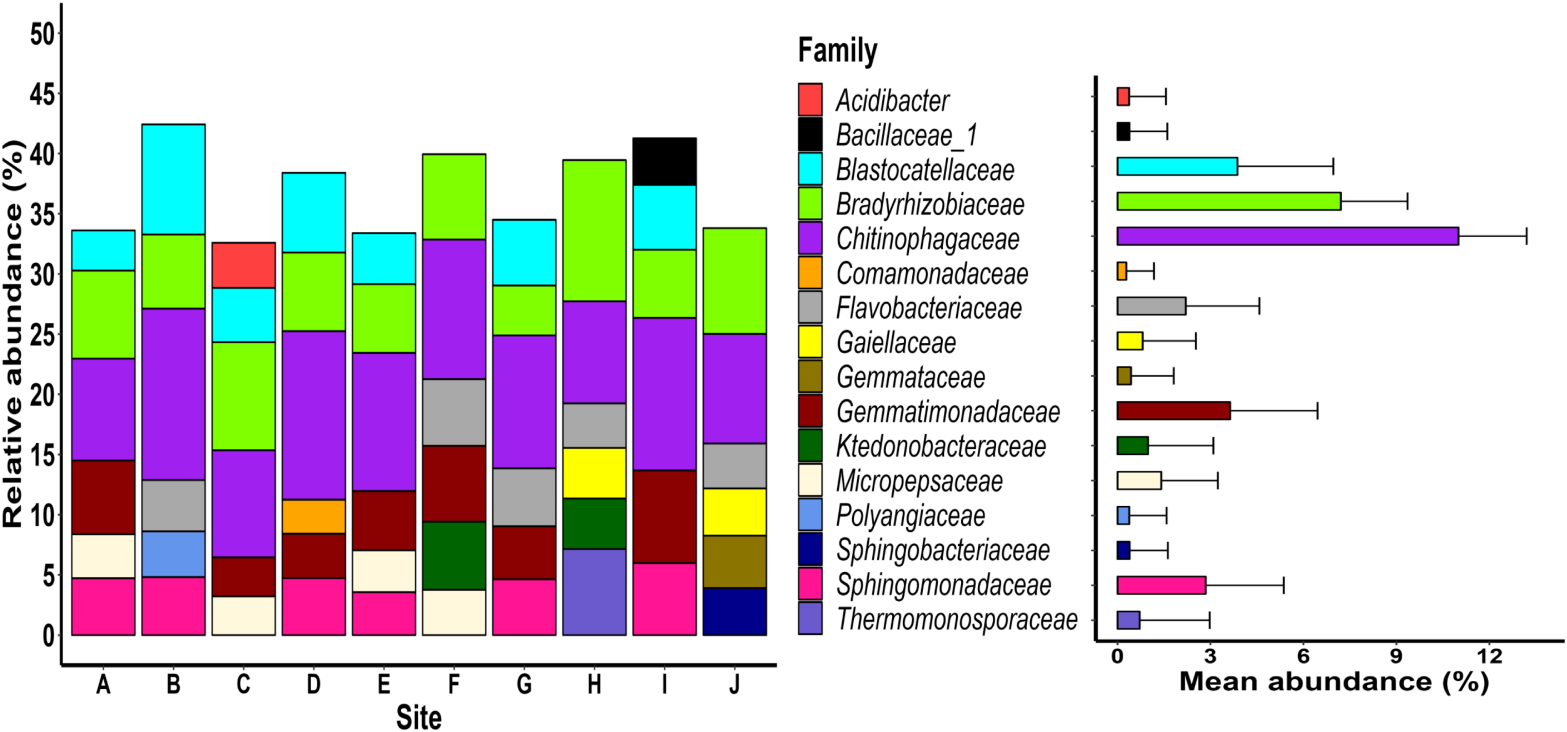
Relative abundance of the six most abundant bacterial families at each site after pooling soil samples from invaded (*Lupinus polyphyllus*) or uninvaded locations. For each family, mean abundance + SD calculated across all soil samples is shown on the right.

We retrieved 9897 bacterial ASVs that were assigned to 25 phyla, 66 classes, 124 orders, 195 families, and 418 genera. The six most common families varied across the ten study sites, but always included *Bradyrhizobiaceae* and *Chitinophagaceae* (Figure 1).

Soil origin had no effect on the three measures of bacterial alpha diversity investigated (Table 1). Moreover, site‐specific slopes were not supported for soil origin (AIC was smaller for the model with a constant slope in all cases), confirming that the effect of soil origin was negligible across sites. Likewise, vegetation cover, legume cover (excluding *L. polyphyllus*), the number of vascular plants, and distance from the road had no relationship with bacterial alpha diversity (Table 1). Based on Nakagawa's *R* ^2^ calculated from the linear mixed models, site identity explained 12%–18% of the variation in the three measures of bacterial alpha diversity (Table 1). Site D had the highest bacterial richness, while site G had the highest bacterial diversity in terms of both Shannon and Inverse Simpson indices.

**TABLE 1:** Results of linear mixed models analysing alpha diversity based on soil bacterial ASVs at ten sites.

	Bacterial richness			Shannon			Inverse Simpson		
	*F*_df,ddf_	*p*	*R* ^2^	*F*_df,ddf_	*p*	*R* ^2^	*F*_df,ddf_	*p*	*R* ^2^
Fixed factors			.055			.022			.013
Soil origin (invaded, uninvaded)	0.155_1,69_	.696		0.100_1,69_	.752		0.464_1,69_	.498	
Vegetation cover (%)	0.969_1,75_	.328		0.771_1,75_	.383		0.746_1,75_	.391	
Legume cover (%)	0.004_1,75_	.950		0.056_1,75_	.813		0.001_1,75_	.765	
Number of plant species	0.023_1,77_	.879		0.366_1,78_	.547		0.861_1,78_	.356	
Distance from the road (m)	2.500_1,55_	.120		0.906_1,63_	.345		0.038_1,61_	.846	
Random factors			.121			.181			.171
Site	NA			NA			NA		

*Note*: Site identity was used as a random factor in all models, NA = not estimated. df and ddf denote the degrees of freedom in the numerator and denominator, respectively. *R* ^2^ refers to the explanatory power of fixed or random factors. Note that the number of vascular plant species was confounded with soil origin and was therefore analysed separately without other fixed explanatory variables (see the methods for details).

### 3.2 Soil bacterial community composition

**FIGURE 2:**
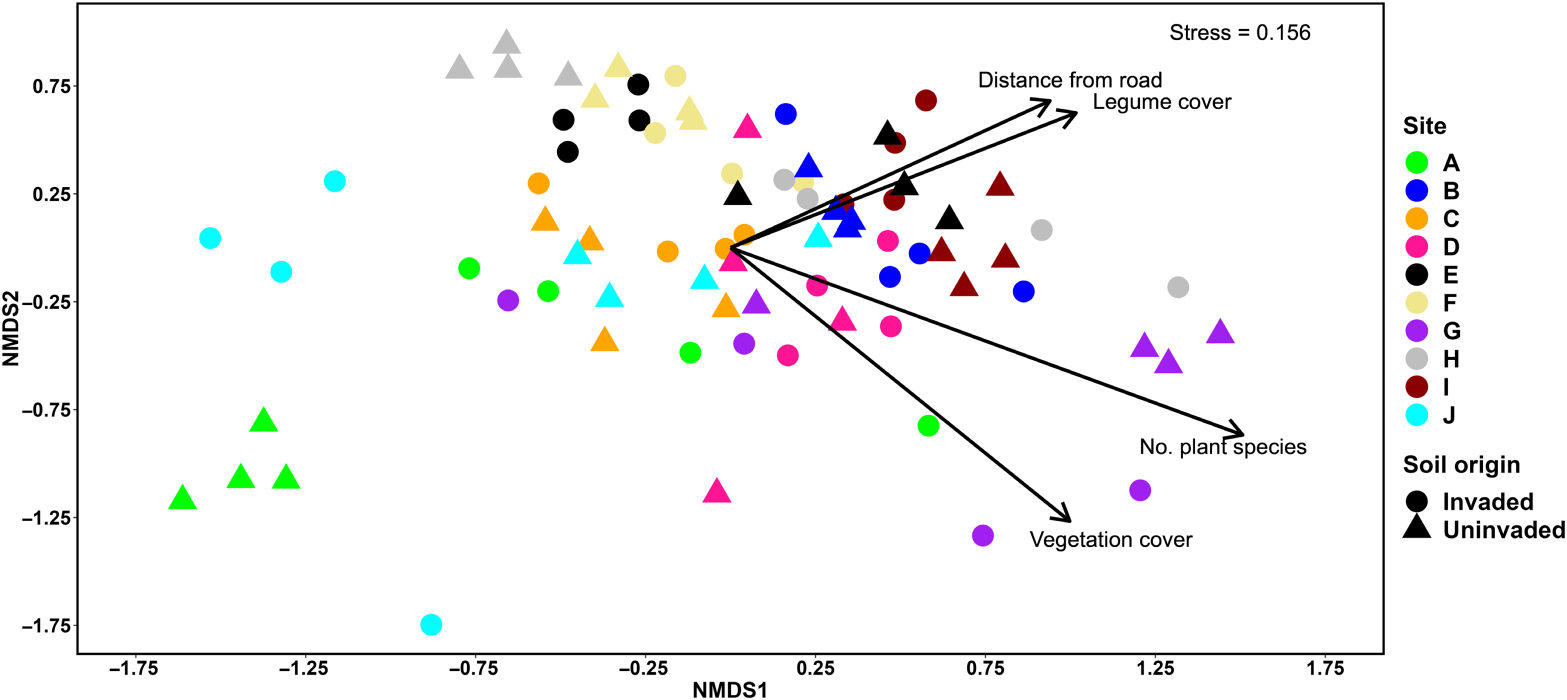
Non‐metric multidimensional scaling (NMDS) ordination of soil bacterial communities in relation to soil origin (invaded by *Lupinus polyphyllus* vs. uninvaded) at ten sites (8 locations per site). The arrows are the fitted vectors for vegetation cover, legume cover after excluding the study species, number of vascular plant species, and distance from the road.

**FIGURE 3:**
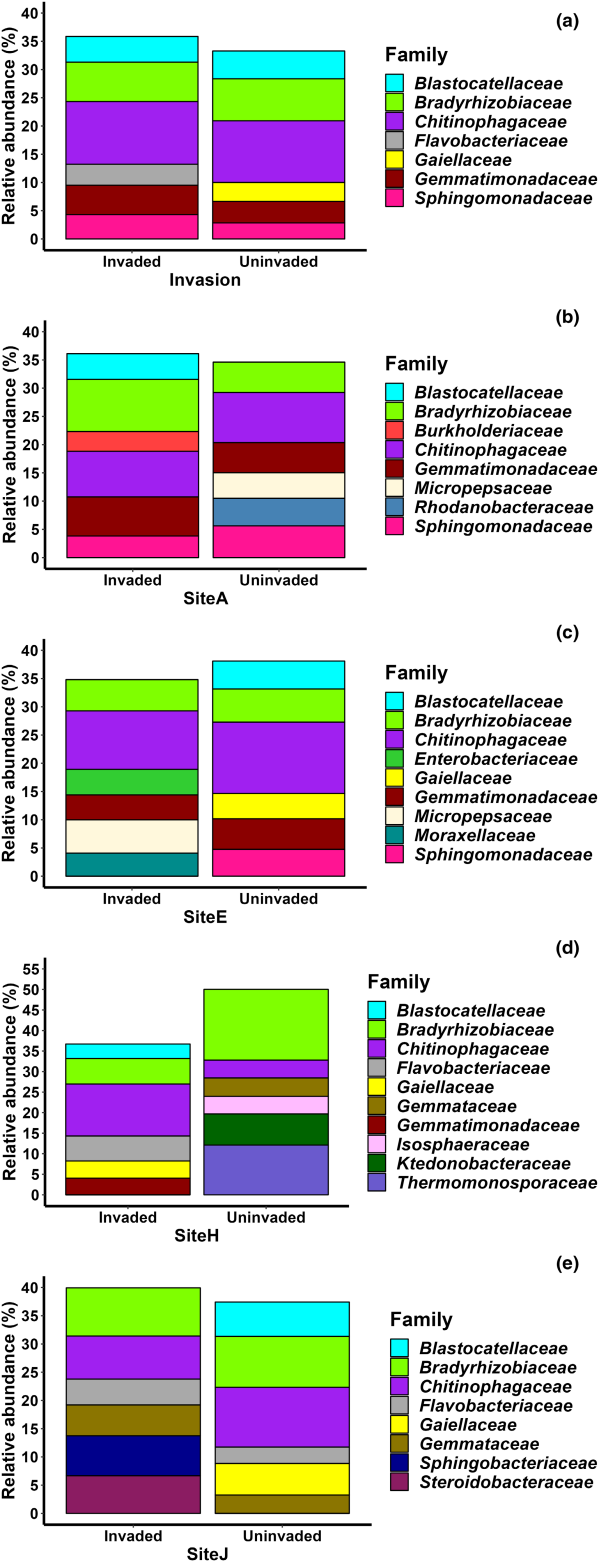
Relative abundance of the six most abundant bacterial families in invaded (*Lupinus polyphyllus*) and uninvaded soils (a) calculated across ten sites and (b–e) at four sites where the bacterial communities cluster differently between soil origins.

The composition of soil bacterial communities differed across the ten sites (Figure 2), with site identity explaining 30.5% of the variation in composition (Table 2). Soil origin per se explained only 1.9% of the variation in bacterial community composition (Table 2) and the top five families (*Chitinophagaceae*, *Bradyrhizobiaceae*, *Blastocatellaceae*, *Gemmatimonadaceae*, and *Sphingomonadaceae*) were equally abundant in invaded and uninvaded soils (Figure 3a). Among the different locations, the main difference in the six most abundant families detected was the presence of family *Flavobacteriaceae* in invaded soils and the presence of family *Gaiellaceae* in uninvaded soils (Figure 3a). The bacterial communities in invaded and uninvaded soils also exhibited the same amount of variation (*F*_1,78_ = 0.460, *p* = .499, that is, the variances were homogeneous). However, soil origin did affect the bacterial communities at some sites (a significant site × soil origin interaction; Table 2). Visual examination of the ordination and dendrogram indicated that the invaded and uninvaded sampling locations clustered differently particularly at sites A, E, H, and J (Figure 2, Figure S1). A closer inspection of the bacterial communities of these four sites revealed that at sites E and J, bacteria belonging to family *Chitinophagaceae* were more abundant in uninvaded soils than in invaded ones (Figure 3c, e), while the opposite was true for site H, where *Chitinophagaceae* were more abundant in invaded soils (Figure 3d). Moreover, at site A, the members of *Bradyrhizobiaceae* dominated invaded soils, whereas at site H they dominated uninvaded soils; at sites E and J, their abundance was unaffected by soil origin (Figure 3b–e). Within each site, certain families were detected in only the invaded or uninvaded soils. The most abundant of these in invaded soils were *Blastocatellaceae* and *Burkholderiaceae* (site A); *Enterobacteriaceae*, *Micropepsaceae*, and *Moraxellaceae* (site E); *Blastocatellaceae*, *Flavobacteriaceae*, *Gaiellaceae*, and *Gemmatimonadaceae* (site H); and *Sphingobacteriaceae* and *Steroidobacteraceae* (site J; Figure 3b–e). On the other hand, the most abundant families detected in uninvaded soils only were *Micropepsaceae* and *Rhodanobacteraceae* (site A); *Gaiellaceae* and *Sphingomonadaceae* (site E); *Gemmataceae*, *Isosphaeraceae*, *Ktedonobacteraceae*, and *Thermomonosporaceae* (site H); and *Blastocatellaceae* and *Gaiellaceae* (site J, Figure 3b–e).

**TABLE 2:** Results of PERMANOVA analysing soil bacterial community composition based on Bray‐Curtis dissimilarities calculated from the relative abundance of bacterial ASVs.

Factors	Df	*F*	*R* ^2^	*p*
Soil origin (invaded, uninvaded)	1	2.404	.019	.002
Site (10 levels)	9	4.383	.305	.001
Vegetation cover (%)	1	1.574	.012	.031
Legume cover (%)	1	2.813	.022	.001
Number of plant species	1	1.839	.023	.004
Distance from the road (m)	1	2.423	.019	.002
Soil origin:Site	9	2.615	.182	.001

*Note*: The number of vascular plant species was confounded with soil origin and was therefore analysed separately, without other explanatory variables.

We conducted indicator species analysis at the ASV level for the four sites where the bacterial communities clustered differently between soil origins (sites A, E, H, J). However, this showed no clear pattern, with the top indicator ASVs varying between soil origins across sites (Table S1). ASVs belonging to family *Bradyrhizobiaceae* were not found to be associated with soil origin, with ASV41 (family *Bradyrhizobiaceae*) being present in both invaded and uninvaded soils (Table S1). At sites A and E, ASVs belonging to class Spartobacteria were often present in invaded soils only (Table S1).

Vegetation cover, legume cover, number of vascular plant species, and distance from the road had minor, although statistically significant, effects on the bacterial community composition, explaining 1.2%, 2.2%, 2.3%, and 1.9% of variation, respectively (Table 2). The number of plant species was highest at site G, vegetation cover was highest in invaded soils at site G, and legume cover was particularly high in uninvaded soils at sites E and I (Figure 2). Distance from the road was greatest for site I (Figure 2). Geographical distance correlated positively with soil bacterial community similarity (*r* = .150, *p* = .004, Mantel test), indicating that geographically close soil bacterial communities were more similar than those that were more distant from each other.

## 4 DISCUSSION

To study the impact of the invasive herbaceous legume *L. polyphyllus* on soil bacterial communities in southwestern Finland where the studied invasions have existed at least since 2010, we carried out amplicon sequencing targeting the bacterial 16S rRNA gene. In contrast to our predictions, we did not observe consistent differences in bacterial alpha diversity or bacterial community composition between invaded and uninvaded soils. The impact of the plant invader on soil bacterial communities varied across sites, but was negligible in the majority of cases. Moreover, total vegetation cover, cover of legumes other than *L. polyphyllus*, the number of vascular plants species, and distance from the nearest road did not appear to be associated with soil bacterial alpha diversity, but did explain some of the variation in bacterial community composition. Our findings suggest that the study species might have a stronger effect on the structure of soil bacterial communities than on their alpha diversity.

The results of a previous meta‐analysis proposed that soil bacterial alpha diversity increases in the presence of invasive plants, particularly for herbaceous invaders that produce allelopathic substances (Torres et al., 2021). Despite the negative allelopathic effects of *L. polyphyllus* on the germination and growth of coexisting herbs (Kalske et al., 2023; Loydi et al., 2015), the current study detected no effect on soil bacterial diversity; the three alpha diversity metrics considered (bacterial richness, Shannon, and Inverse Simpson indices) did not differ between invaded and uninvaded soils. The negligible effect of this plant invader on soil bacterial alpha diversity is somewhat unexpected, given the fact that *L. polyphyllus* has been reported to reduce vascular plant species richness independently of the amount of time since its invasion (Prass et al., 2022). Such a reduction in plant species richness has been documented also at invaded sites in the study area (Ramula & Pihlaja, 2012). However, the minor role we detected for *L. polyphyllus* in modifying soil microbial alpha diversity is in line with findings from another meta‐analysis that reported heterogeneous but limited impacts of plant invaders on soil microbial alpha diversity across multiple ecosystems (Custer & van Diepen, 2020). In the present study, site identity explained considerably more of the variation (12%–18%) in measures of bacterial alpha diversity than the plant invader, with site G having the highest bacterial diversity. That site tended to also have the greatest number of vascular plant species.

Similar to findings from a woody legume, the Australian acacia (Keet et al., 2021), we discovered that the composition of soil bacterial communities was more responsive to the presence of *L. polyphyllus* than soil bacterial alpha diversity. However, the responses of the soil bacterial communities were inconsistent across sites, which did not support our hypothesis that the leguminous invader would increase the relative abundance of bradyrhizobia, that is, members of family *Bradyrhizobiaceae*. Although invaded soils in this study did not appear to contain a higher relative abundance of *Bradyrhizobiaceae* or a higher number of indicator ASVs belonging to this family, these bacteria were among the most abundant in both invaded and uninvaded soil samples. Besides *L. polyphyllus*, which was only present in the invaded locations, other common legumes at our sampling sites were the red clover (*Trifolium pratense*), the zigzag clover (*T. medium*), the white clover (*T. repens*), and the common vetch (*Vicia sativa*). As these four legumes form nodules mostly in symbiosis with *Rhizobium leguminosarum* (Duodu et al., 2006; Mothapo et al., 2013), they cannot explain the high abundance of *Bradyrhizobiaceae* in the uninvaded soils. Instead, it is possible that the high abundance of *Bradyrhizobiaceae* across the study sites is due to the presence of free‐living bradyrhizobia (non‐symbiotic and/or non‐diazotrophic) that inhabit the roots of non‐leguminous plants and even the guts of earthworms and insects in the soil (Degli Esposti & Martinez Romero, 2017; Rivas et al., 2004; Thakuria et al., 2010; Wasai‐Hara et al., 2020). To date, such free‐living bradyrhizobia have been found in soils in European grasslands and North American forests (Jones et al., 2016; VanInsberghe et al., 2015). With our study design, it was not possible to determine the exact function of bradyrhizobia in the uninvaded soils, but future sequencing studies focused on a different gene, such as *rpoB* (Mousavi et al., 2022), would enable finer‐scale taxonomic identification and shed light on the potential roles of these bacteria.

The other factors we investigated‐vegetation cover, legume cover, the number of vascular species, and distance from the road‐had no effect on soil bacterial alpha diversity, but were all slightly associated with soil bacterial community composition. These findings do not provide resounding support for the hypothesis that plant functional groups play a prominent role in determining soil microbial communities (Dassen et al., 2017; Delgado‐Baquerizo et al., 2018; Hui et al., 2017). However, the cover of legumes other than *L. polyphyllus* was low in the present study (mean = 3.1%, range: 0%–52%), which might explain its negligible role in shaping soil bacterial alpha diversity. Similar to previous studies reporting the importance of soil type and soil properties in shaping soil bacterial structure (Lauber et al., 2008; Rodrigues et al., 2013; Xue et al., 2018), we observed that bacterial community compositions differed greatly across the ten study sites, with only two bacterial families (*Chitinophagaceae* and *Bradyrhizobiaceae*) being particularly abundant at all ten sites. Both of these families are ubiquitous in a variety of boreal and temperate soils (Fernandez‐Gnecco et al., 2022; Hartmann et al., 2012; Wolińska et al., 2018), with the family *Chitinophagaceae* (phylum *Bacteroidetes*) being involved in the degradation of organic matter (Bailey et al., 2013). Such among‐site variation in bacterial communities was to be expected because the study sites represented four different habitats and different soil types. Bacterial community structure is also affected by both the physical and chemical properties of soil (e.g. Burns et al., 2015; Ehrenfeld et al., 2005), but as these were not considered in the present study, the interpretation of our results in this regard is limited. Moreover, the rhizosphere soil in the uninvaded locations were collected from different dominant plant species, which is likely to have contributed to the among‐site variation in the soil bacterial communities (Burns et al., 2015) and may have confounded our results. Similarly, the developmental stage of the sampled plants (vegetative or flowering in the present study) might have also contributed to the among‐site variation in soil bacterial communities (e.g. Ajilogba et al., 2022; Chaparro et al., 2014). Finally, we have no information on the past agricultural history (if any) and soil legacies of the sampling sites, which might also have contributed to some of the observed differences. The effect of the final factor we investigated, distance from the road, is more easily explained: it is likely due to a combination of traffic pollutants (e.g. heavy metals) and/or the use of road de‐icing salt in winters, which are both known to alter soil bacterial communities (e.g. Ke et al., 2013; Tan et al., 2022).

Our results suggest that in the area studied here, the invasive legume *L. polyphyllus* may not be a key driver determining soil bacterial alpha diversity or bacterial community composition in sites that were invaded more than a decade ago. As we focused on bacterial communities only, we cannot rule out the possibility that *L. polyphyllus* might still affect soil fungal communities. The generality of our findings may also be limited by the fact that the exact age of the studied invasions is not known. The effects of plant invasions on resident communities have been suggested to change over time due to, for example, the accumulation of soil pathogens (Flory & Clay, 2013), and such changes can occur even after a decade. For example, the benefits of soil microbial communities to the invasive legume *Vicia villosa* were lower at sites that had been invaded more than a decade earlier compared to sites with more recent invasions (Lau & Suwa, 2016). In another study, the effects of invasive grasses on soil nitrogen‐mineralisation rates were considerably reduced over a 20‐year time period (Yelenik & D'Antonio, 2013). Moreover, microbial dispersal in soil can weaken soil microbial legacies, making them hard to detect even after just a few years (Wang & Allison, 2021). Here, all studied invasions have existed at least since 2010 (Ramula, 2014), and therefore, further studies would be necessary to determine if small belowground effects are detectable in more recent invasions.

## 5 CONCLUSIONS

When taken together with previous research, our findings indicate that the effects of invasive plants on soil microbiota might be species‐specific (Stefanowicz et al., 2016) as well as specific to local site conditions (Keet et al., 2021). We observed that site identity greatly affected both the alpha diversity and community composition of soil bacteria, with vegetation cover, the cover of non‐invasive legumes, the number of plant species, and distance from the nearest road also modifying bacterial community structure. Although the invasion of *L. polyphyllus* appeared to have a negligible effect on soil bacterial alpha diversity, it was associated with inconsistent shifts in the composition of soil bacterial communities at some sites. Such shifts in soil bacterial community composition can lead to changes in the aboveground communities and ecosystem function (e.g. Coats & Rumpho, 2014; Milanović et al., 2020; Wardle et al., 2004), with potentially important ramifications.

## AUTHOR CONTRIBUTIONS

**Seyed Abdollah Mousavi:** Conceptualization (lead); data curation (lead); formal analysis (lead); investigation (lead); methodology (lead); visualization (lead); writing – original draft (lead); writing – review and editing (supporting). **Satu Ramula:** Conceptualization (supporting); data curation (supporting); formal analysis (supporting); funding acquisition (lead); methodology (supporting); supervision (lead); visualization (supporting); writing – original draft (supporting); writing – review and editing (lead).

## CONFLICT OF INTEREST STATEMENT

There are no competing interests to declare by the authors.

## Supporting information


Figure S1.
Table S1.

## Data Availability

Raw data analysed in this study are deposited in the Sequence Read Archive (SRA) database (accession no. SRR23356334–SRR23356411, under the BioProject PRJNA932185). The data used for the analyses are deposited in Dryad (DOI: 10.5061/dryad.8sf7m0cwr).
