# Supplementary material for: The invasive legume Lupinus polyphyllus has minor site‐specific impacts on the composition of soil bacterial communities
Source: Ecol Evol. 2024 Feb 13;14(2):e11030. doi: 10.1002/ece3.11030 (PMC10864723; doi:10.1002/ece3.11030)
Supplement: Supplementary file 1 — Figure S1. Table S1. [file ECE3-14-e11030-s001.docx]

**The invasive legume *Lupinus polyphyllus* has minor site-specific impacts on the composition of soil bacterial communities**

Seyed Abdollah Mousavi^1^*, Satu Ramula^1^

^1^Department of Biology, University of Turku, 20014 Turku, Finland

* **Correspondence:** [s.abdollah.mousavi@gmail.com](mailto:s.abdollah.mousavi@gmail.com)

ORCID to SAM: 0000-0001-9230-1481, SR: 0000-0001-7795-0352

**Figure S1** Cluster analysis (UPGMA) of soil bacterial communities in relation to soil origin (invaded by *Lupinus polyphyllus* vs. uninvaded) at ten sites (A-J) in southwestern Finland. Bray-Curtis dissimilarities are calculated from mean relative bacterial abundances, ASVs (N = 4 samples per branch), and sites are indicated with different colours.

**Table S1** Top ten indicator bacterial ASVs, determined to the lowest taxonomic level, and mean relative abundance ± SD in invaded (*Lupinus polyphyllus*) and uninvaded soils at four sites where the bacterial communities cluster differently between the two locations. The taxonomic levels shown are k = kingdom, p = phylum, c = class, o = order, f = family and g = genus. Bradyrhizobial ASVs are in bold.

| **Site A, Invaded** | **Site A, Uninvaded** |
| --- | --- |
| ASV1, c_Spartobacteria, 1.29 ± 0.71 | ASV170, f_Chitinophagaceae, 1.79 ± 0.63 |
| ASV2, c_Spartobacteria, 0.61 ± 0.24 | ASV247, g_Arthrobacter, 0.95 ± 0.22 |
| ASV4, c_Spartobacteria, 0.77 ± 0.35 | ASV302, c_Acidobacteria_Gp6, 0.22, 0.07 |
| ASV38, g_Stenotrophobacter, 0.41 ± 0.35 | ASV321, c_Acidobacteria_Gp6, 0.42 ± 0.25 |
| **ASV41, f_Bradyrhizobiaceae, 0.56 ± 0.12** | ASV323, g_Ureibacillus, 1.07 ± 1.00 |
| ASV44, g_Flavobacterium, 0.37 ± 0.10 | ASV329, f_Chromobacteriaceae, 1.00 ± 0.59 |
| ASV50, c_Gammaproteobacteria, 0.12 ± 0.05 | ASV353, c_Betaproteobacteria, 0.99 ± 0.27 |
| ASV72, f_Sterolibacteriaceae, 0.44 ± 0.06 | ASV363, g_Planifilum, 0.90 ± 0.25 |
| ASV81, g_Lacipirellula, 0.39 ± 0.14 | ASV370, c_Acidobacteria_Gp10, 0.83 ± 0.75 |
| ASV106, g_Paraburkholderia, 0.68 ± 0.15 | ASV382, g_Sphingomonas, 0.98 ± 0.55 |
| **Site E, Invaded** | **Site E, Uninvaded** |
| ASV28, c_Spartobacteria, 0.36 ± 0.11 | **ASV7, g_Bradyrhizobium, 0.76 ± 0.25** |
| ASV32, c_Spartobacteria, 0.53 ± 0.23 | ASV29, c_Acidobacteria_Gp6, 0.69 ± 0.40 |
| ASV43, c_Spartobacteria, 0.33 ± 0.14 | ASV39, c_Acidobacteria_Gp4, 1.35 ± 1.49 |
| ASV74, c_Spartobacteria, 1.35 ± 0.66 | ASV42, c_Acidobacteria_Gp7, 0.31 ± 0.09 |
| ASV75, c_Acidobacteria_Gp1, 0.55 ± 0.05 | ASV61, g_Hyphomicrobium, 0.38 ± 0.02 |
| **ASV103, f_Bradyrhizobiaceae, 0.31 ± 0.09** | ASV84, c_Spartobacteria, 0.25 ± 0.11 |
| ASV132, c_Acidobacteria_Gp4, 0.26 ± 0.09 | ASV85, c_Acidobacteria_Gp6, 0.37 ± 0.24 |
| ASV144, c_Acidobacteria_Gp6, 0.23 ± 0.14 | ASV93, f_Chitinophagaceae, 0.53 ± 0.10 |
| ASV186, g_Acinetobacter, 0.47 ± 0.27 | ASV105, c_Acidobacteria_Gp6, 0.21 ± 0.04 |
| ASV193, k_Bacteria, 0.29 ± 0.04 | ASV129, c_Betaproteobacteria, 0.53 ± 0.11 |
| **Site J, Invaded** | **Site J, Uninvaded** |
| ASV6, k_Bacteria, 8.44 ± 9.50 | ASV63, f_Steroidobacteraceae, 0.32 ± 0.21 |
| ASV16, f_Chitinophagaceae, 1.16 ± 0.33 | ASV82, o_Candidatus_Solibacter, 0.54 ± 0.27 |
| ASV31, g_Stenotrophobacter, 0.38 ± 0.14 | ASV141, o_Acidimicrobiales, 0.31 ± 0.06 |
| ASV38, g_Stenotrophobacter, 0.26 ± 0.08 | ASV291, o_Rhodospirillales, 0.30 ± 0.18 |
| ASV40, p_Proteobacteria, 0.32 ± 0.15 | ASV364, c_Acidobacteria_Gp2 ± 0.95, 0.13 |
| ASV61, g_Hyphomicrobium, 0.23 ± 0.06 | ASV385, p_Proteobacteria, 0.18 ± 0.10 |
| ASV62, g_Sphingomonas, 0.27 ± 0.12 | ASV390, c_Acidobacteria_Gp1 ± 0.47, 0.20 |
| ASV64, g_Arenimonas, 0.29 ± 0.07 | ASV398, c_Acidobacteria_Gp1 ± 0.35, 0.23 |
| ASV84, c_Spartobacteria, 0.41 ± 0.25 | ASV400, f_Gemmataceae, 0.29 ± 0.10 |
| ASV89, g_Pseudolabrys, 0.39 ± 0.15 | ASV410, c_Spartobacteria, 0.70 ± 0.09 |
| **Site H, Invaded** | **Site H, Uninvaded** |
| ASV481, k_Bacteria, 0.43 ± 0.08 | **ASV41, f_Bradyrhizobiaceae, 0.56 ± 0.31** |
| ASV1746, c_Betaproteobacteria, 0.18 ± 0.11 | ASV47, c_Acidobacteria_Gp4, 0.99 ± 0.82 |
| ASV27, c_Acidobacteria_Gp6, 0.78 ± 0.22 | ASV56, c_Betaproteobacteria, 0.51 ± 0.36 |
| ASV269, g_Paraburkholderia, 0.48 ± 0.11 | ASV59, p_Verrucomicrobia, 0.54 ± 0.30 |
| - | ASV61, g_Hyphomicrobium, 0.49 ± 0.25 |
| - | ASV94, c_Acidobacteria_Gp6, 0.15 ± 0.06 |
| - | ASV100, c_Acidobacteria_Gp6, 0.42 ± 0.20 |
| - | ASV107, g_Opitutus, 0.30 ± 0.17 |
| - | ASV110, c_Spartobacteria, 0.47 ± 0.22 |
| - | ASV164, c_Acidobacteria_Gp4, 0.14 ± 0.09 |
